# Small-molecule and mutational analysis of allosteric Eg5 inhibition by monastrol

**DOI:** 10.1186/1472-6769-6-2

**Published:** 2006-02-27

**Authors:** Zoltan Maliga, Timothy J Mitchison

**Affiliations:** 1 MPI-CBG, Pfotenhauer Strasse 108, 01307 Dresden, Germany; 2 Department of Systems Biology, Harvard Medical School, Boston, Massachusetts, USA

## Abstract

**Background:**

A recent crystal structure of monastrol in a ternary complex with the kinesin Eg5 motor domain highlights a novel, induced-fit drug binding site at atomic resolution. Mutational obliteration of the monastrol binding site results in a monastrol-resistant, but otherwise catalytically active Eg5 motor domain. However, considering the conformational changes at this site, it is unclear what specific interactions stabilize the interaction between monastrol and the Eg5 motor domain.

**Results:**

To study the molecular complementarity of the monastrol-Eg5 interaction, we used a combination of synthetic chemistry and targeted mutations in Eg5 to measure the contribution of specific contacts to inhibition of Eg5 in vitro and in cultured cells. Structure-activity data on chemical derivatives, sequence analysis of Eg5 homologs from different species, and the effect of mutations near the drug binding site were consistent with the crystal structure.

**Conclusion:**

The mechanism of monastrol revealed by our data rationalizes its specificity for Eg5 over other kinesins and highlights a potential mechanism of drug resistance for anti-cancer therapy targeting this site in Eg5.

## Background

Kinesins are a diverse family of microtubule-based motor proteins important for intracellular transport and cell division in all eukaryotes [1,2]. Genetic and biochemical dissection of kinesin function implicates specific kinesins in the trafficking of organelles [3], signaling complexes [4], and vesicular cargo [5]. During mitosis and cytokinesis, kinesins are essential for microtubule dynamics regulation, assembly and maintenance of bipolar spindles, and accurate chromosome segregation [6]. Assessing the precise contributions of kinesins to highly dynamic processes during both interphase and mitosis is challenging. Genetic tools such as siRNA are general and specific, but lack temporal resolution and reversibility necessary for detailed analysis of dynamic processes. Reversible, small molecule inhibitors of both microtubule- and actin-based motors are proving to be invaluable tools with which to study their functions during cell division [7,8]. Monastrol, a specific inhibitor of the BimC class kinesin Eg5 (also called kinesin-5 or kinesin spindle protein, KSP) [7], has permitted more critical analyses of Eg5 function during spindle assembly [9,10] and as a reversible agent to synchronize cells in metaphase [8]. Furthermore, inhibitors of Eg5 and other mitotic kinesins are plausible anti-cancer drugs now under development and testing [11,12]. They work by disrupting the mitotic spindle, arresting cancer cells in mitosis, and thus triggering apoptosis [13].

BimC class kinesins are widely required for bipolar spindle assembly during mitosis and meiosis. They are homotetrameric, plus-end directed kinesins that associate with the spindle during mitosis [14-16] and their inhibition or removal generally results in spindle collapse [7,17,18]. Spindles are dynamic, bipolar arrays of microtubules that are maintained in part by a balance of forces between oppositely directed motor proteins [19,20], and it is likely that Eg5 provides the forces that drives the two spindle poles apart from each other. Monastrol reversibly inhibits microtubule gliding by Eg5 and causes spindle collapse in cells [7]. Eg5 supports anti-parallel sliding of microtubules *in vitro *[21] and during poleward flux in Xenopus extract spindles, a process completely inhibited by monastrol [22]. Biochemical analysis of microtubule binding [23] and gliding by Eg5 *in vitro *[24] indicates monastrol induces microtubule release and therefore complete loss of Eg5 function as a microtubule cross-linker and motor.

**Figure 1:**
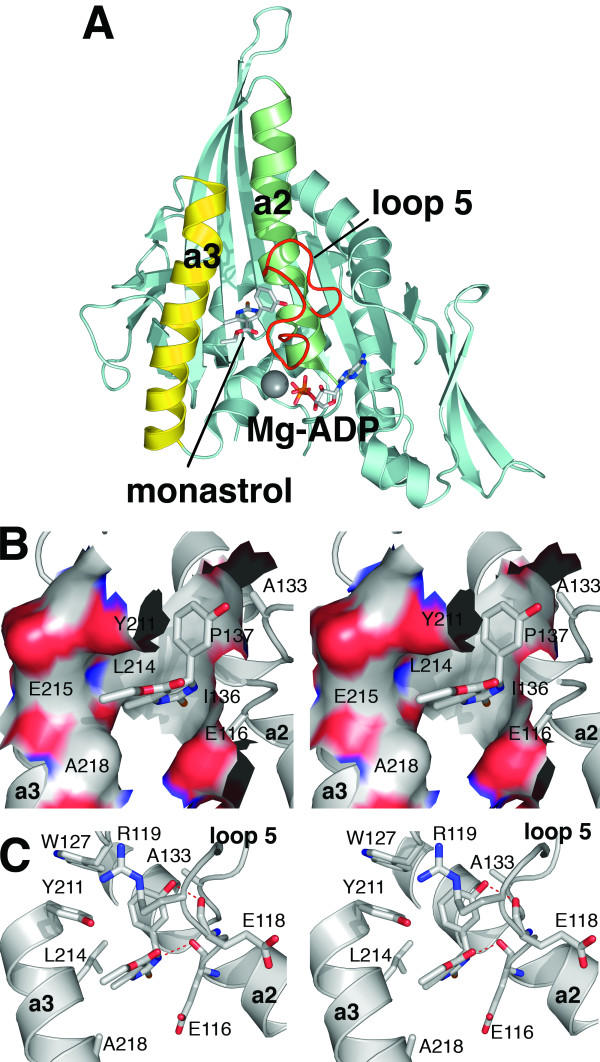
**Ternary complex of Eg5 motor domain with monastrol and ADP-Magnesium**. Ternary complex of Eg5 motor domain with monastrol and ADP-Magnesium. (A) Ribbon diagram of the Eg5 motor domain (pale blue) with the nucleotide binding site facing down, containing ADP-Magnesium (adapted from PDB: 1Q0B). Monastrol is bound 12 Å from the nucleotide binding pocket, in a pocket formed by residues in α2 (green, labeled 'a2'), α3 (yellow, labeled 'a3') and loop 5 (red), a structural feature interrupting α2. (B) Stereoscopic view of monastrol binding site to illustrate the hydrophobic dihydropyrimidine binding surface between α2 and α3 formed primarily by the side chains of amino acid residues E116, I136, P137, Y211, L214, and A218. The van der Waals contact surface of Eg5 is labeled with its corresponding amino acid residue and is colored gray for carbon, red for oxygen, and blue for nitrogen. (C) The hydroxyphenyl ring is oriented perpendicular to the plane of the dihydropyrimidine ring in a hydrophobic pocket bounded by the side chains of amino acid residues R119, W127, A133, and Y211. Two potential hydrogen bonding interactions between monastrol and the backbone carbonyls oxygens of amino acid residues E116 and E118 are highlighted in red. Distances from monastrol N to E116 and O to E118 are 2.82 Å and 2.73 Å, respectively.

The co-crystal structure of Eg5 bound to monastrol reveals the drug binding site in atomic detail. Monastrol binds in a hydrophobic, induced-fit pocket between two non-conserved features of kinesin motor domains, loop 5 and alpha-helix 3 (α3) (Figure 1A). Upon drug binding, α3 moves 1Å relative to alpha-helix 2 (α2) and loop 5 folds onto the drug binding site [25]. Specific hydrophobic (Figures 1B and 1C) and polar (Figure 1C) interactions appear in the crystal structure, but it is unclear how they contribute to drug binding. There is direct [12] or biochemical evidence that at least two other structural classes of Eg5 inhibitors target this site, reducing dynamics of α3 and loop 5 [26]. Considering the changes in the structure of this site, it is unclear how these interactions contribute to drug binding, specificity, and inhibition of Eg5.

Guided by the atomic structure of the Eg5-ADP-monastrol ternary complex, we used a combination of synthetic chemistry, targeted mutagenesis, and protein biochemistry to characterize the interaction between the Eg5 motor domain and monastrol at the atomic level. Initially, we confirm that the structure is consistent with species-specificity of monastrol in cells. A structure-activity relationship for monastrol and mutation of target binding residues tests the importance of individual amino acid residues and chemical substituents to the protein-drug interaction.

## Results

### Confirmation of crystal structure

We crystallized the monastrol-ADP-Eg5 motor complex, and solved its structure, independent of the published work [25] to 1.8 Å resolution. Our structure confirms the conformational changes in the Eg5 motor domain induced by monastrol binding in protein crystals obtained using distinct precipitant conditions. The details of crystallization and atomic structure determination are available as supplemental information (see Additional files 1 and 2).

### Structure-activity relationship of monastrol derivatives *in vitro *and in cultured cells

**Figure 2:**
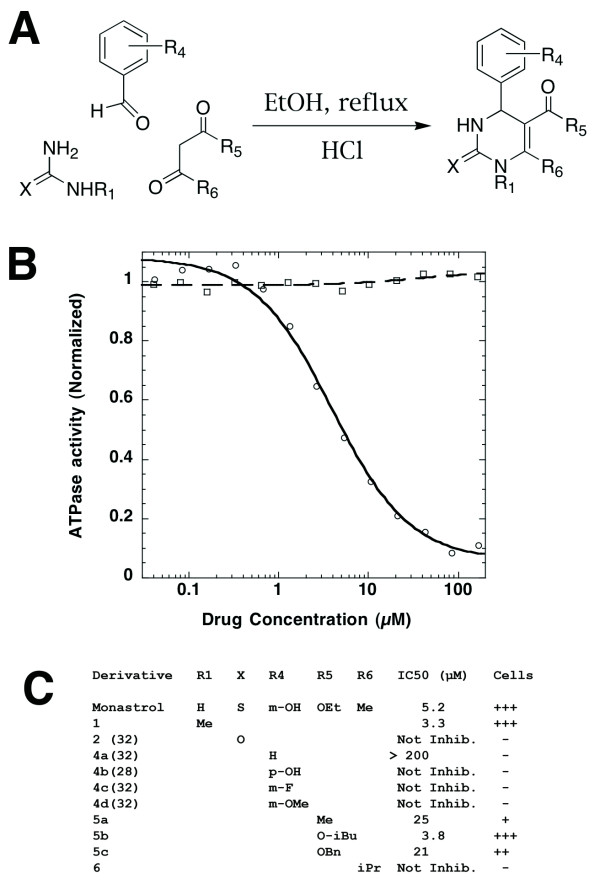
**Structure-activity relationship of monastrol derivatives**. (A) Scheme depicting the Biginelli condensation, a one-pot reaction for preparing monastrol derivatives [40]. (B) Monastrol (circles), but not derivative 2 (squares), inhibits ATP hydrolysis by 500 nM hEg5-367H in KC25 buffer supplemented with 1 mM ATP-KOH (pH 7.0). The IC_50 _for monastrol is 5.2 +/- 0.4 μM. Each data point is the average of three independent experiments. (C) Summary of SAR results for monastrol derivatives inhibiting the catalytic activity of the Eg5 motor domain and inducing monoaster formation in tissue culture cells, as described [28]. IC_50 _for each inhibitor was measured as described in part B. In cell-based assays, the potency of each derivative was obtained by treating BS-C-1 cells as described in the methods section, and manually counting mitotic cells to yield a score as follows: the drug concentration with roughly equal monastral and bipolar spindles, of < 50 μM (+++), weak compounds 50 to 200 μM (+ or ++), and inactive (-) compounds displayed no effect at 200 μM. Each cell-based assay was performed in duplicate.

We synthesized a variety of monastrol derivatives using the Biginelli cyclo-condensation (Figure 2A) to determine the small-molecule structure-activity relationship (SAR) for inhibition of purified, recombinant Eg5 motor domain *in vitro *and monoaster formation in tissue culture cells. Overall, SARs for *in vitro *and *in vivo *activity are tightly correlated, and consistent with the crystal structure of the monastrol binding site. We found that any modification of R_4 _abolishes monastrol activity, supporting its role as a hydrogen bond donor. Substituting oxygen for sulfur at position 2 also abolishes activity (Figure 2B), presumably due to loss of favorable hydrophobic packing interactions with Eg5. Introducing a bulky isopropyl group at R_6 _abolishes activity by physically clashing with the side chains of A218 and E215. By contrast, methylation of position 1 and a range of substitutions at position 5 do not abolish drug activity (Figure 2C). This observation strongly supports a view that the binding mode observed by X-ray crystallography is relevant to biochemical inhibition, and also the view than monopolar spindle assembly in cells is due to Eg5 inhibition.

### Monastrol is specific for vertebrate Eg5 homologs

**Figure 3:**
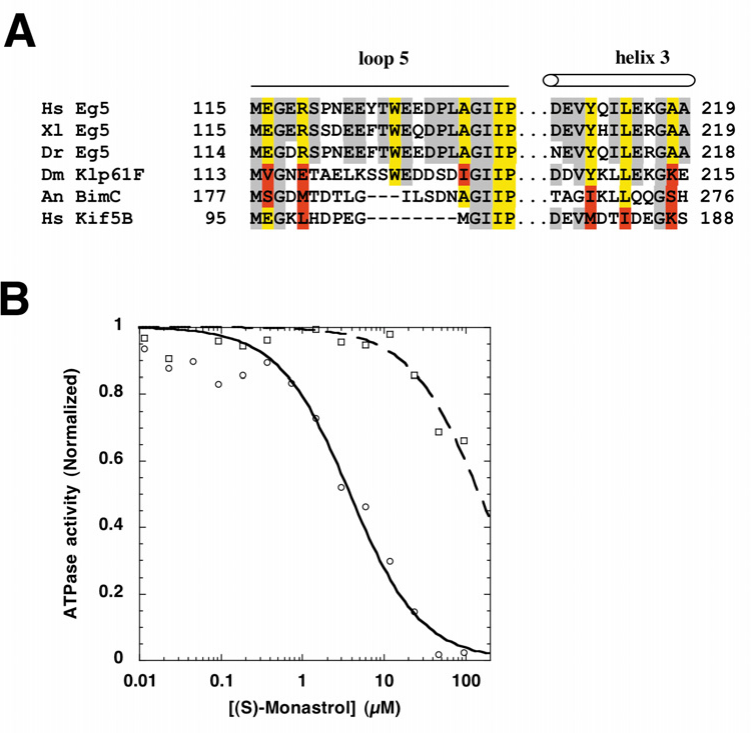
**Sequence requirements for monastrol binding to Eg5**. (A) Amino acid sequence alignment of residues near the monastrol binding site in Eg5 homologs from *Homo sapiens*, *Xenopus laevis*, *Danio rerio, Drosophila melanogaster*, *Aspergillus nidulans *and the corresponding residues in human conventional kinesin (Kif 5B). Residues that are conserved among monastrol-sensitive species [7, 27, 28] are highlighted in yellow in all homologs where they are identical. Amino acid side chains expected to occlude the drug binding site are highlighted in red. (B) Measuring ATP hydrolysis by 400 nM hEg5-367H (circles) or 620 nM Klp61F-364H (squares) in the presence of 1 mM ATP over a range of (S)-monastrol concentrations. Each data point is the average of three independent experiments. The IC_50 _for (S)-monastrol for Eg5 and Klp-61F are 6.1 +/- 0.7 and 230 +/- 4 μM, respectively.

Monastrol binds a hydrophobic pocket between loop 5 and α3 that is poorly conserved among kinesins, resulting in specificity for vertebrate Eg5 homologs. The amino acid side chains that contact monastrol are conserved in human Eg5 and its homologs from *X. laevis *and *D. rerio*, but not *D. melanogaster *Klp-61F, *A. nidulans *BimC or human conventional kinesin (Figure 3A). Monastrol is known to inhibit human and Xenopus Eg5 [7,9], but not BimC [27]. To test its effect on *D. rerio *Eg5 and Klp-61F, we treated AB9 and KC_167 _cells with monastrol. We observed mitotic arrest in *D. rerio*, and normal, bipolar spindles in *Drosophila *cells (data not shown). Since Klp-61F is required for spindle bipolarity [6], we interpret this result to mean that Klp-61F is unaffected by monastrol. To obtain unambiguous evidence, we cloned the motor domain of Klp-61F and subjected the recombinant protein to ATPase assays in the presence or absence of microtubules. As shown in Figure 3B, microtubule-stimulated ATP hydrolysis by the Klp-61F motor domain is weakly inhibited by monastrol (Figure 3B), consistent with the monastrol-insensitivity of KC_167 _cells. Thus, species cross-reactivity of monastrol is consistent with the drug binding site observed in the crystal structure. Monastrol, or any other drugs that bind at the same site, will probably be inactive in invertebrate cells, and should be used as research tools in such cells only with the greatest of caution. That said, it might be interesting to introduce drug sensitive vertebrate Eg5 into invertebrate cells for study.

### Mutational analysis of monastrol binding site

**Figure 4:**
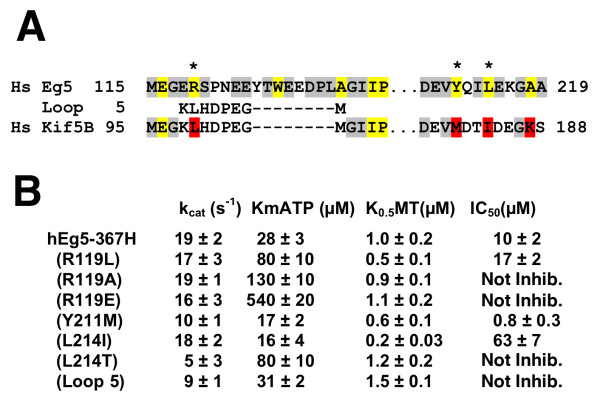
**Mutagenesis of the monastrol binding site in human Eg5**. (A) Design of mutations in the human Eg5 motor domain. Amino acid side chains that are not conserved between Eg5 and conventional kinesin were targeted, either introducing chimeric or rationally designed mutations (asterix for targeted point mutants) to test the contribution of residues to the drug-sensitivity of Eg5. (B) Steady-state enzymatic parameters for engineered human Eg5 motor domain mutants. Steady-state ATP hydrolysis by Eg5 was measured using an NADH-enzyme coupled assay varying the concentration of ATP, microtubules, or monastrol to obtain the microtubule concentration for half-maximal stimulation (K_0.5_MT), the maximal rate of microtubule-stimulated ATP hydrolysis (k_cat_), and the concentration of monastrol that inhibits basal ATP hydrolysis by 50 % (IC_50_), as described [28].

To further test the sequence requirements for monastrol binding and specificity, we introduced mutations into the motor domain of human Eg5. The mutations replaced amino acid residues lining the monastrol-binding pocket with the corresponding residues from human conventional kinesin (Figure 4A), or were engineered to test if hydrophobic interactions are required for drug binding. Steady-state microtubule-stimulated ATP hydrolysis by the purified recombinant motor domain provided a convenient measure of Eg5 activity and inhibition by monastrol [28]. The value for k_cat _is higher than in some publications [23,25], consistent with different buffer concentration, which we have previously observed have a large effect not only on K_0.5_MT [27], but also k_cat _(unpublished data). We infer that the Eg5 motor domain is remarkably tolerant of mutations at the monastrol binding site, as all mutant protein constructs displayed nearly wild-type catalytic activity. A few mutations had a marked effect on the enzymatic parameters of Eg5. In particular, mutating R119 elevated K_m_ATP and L214I, a remarkably conserved mutation, lowered K_0.5_MT. A chimera mutation replacing loop 5 of Eg5 with that of conventional kinesin was monastrol-resistant (Figure 4B), consistent with a related study [26].

All of the point-mutants, with the exception of Y211M, decreased the drug sensitivity of Eg5. Y211M actually increased the monastrol sensitivity of Eg5, perhaps because flexibility of the methionine side chain accommodates a more favorable hydrophobic interaction with the drug. Hydrophobic packing between the thiourea and L214 appears to be crucial for drug binding, illustrated by the dramatic effect of even a subtle L214I mutation on IC_50_. L214T mutant was resistant to monastrol, as would be expected, but also to derivative 2 (data not shown) with a compensating hydrophilic urea. Hydrophobic contacts from R119 are required for monastrol binding, but electrostatic interactions also appear important (Figure 4B). In summary, our mutational changes near the binding site are consistent with the Eg5-monastrol structure and provide a number of catalytically active mutants of Eg5 that might be useful to examine spindle assembly and drug resistance in Eg5 by expression in cells.

## Discussion

Monastrol is a convenient reagent for mitosis research, but its specificity hitherto has not been established. Experiments designed to probe the role of Eg5 in the mitotic spindle and post-mitotic neurons are predicated on the assumption that monastrol is a specific inhibitor of Eg5 [29,30]. It is reassuring to note that no kinesin protein contains sequences in loop 5 and α3 that are part of the monastrol binding site. Loop 5 is highly divergent among different kinesin classes [25], and the sequence of α3 near the monastrol binding site is not conserved. Indeed, most kinesins project bulky amino acid side chains into the putative monastrol binding pocket that would preclude drug binding (Figure 3A). Thus, monastrol should be highly selective among kinesins. However, we note it could easily target unexpected proteins. Its affinity is fairly low by drug standards, and it is often used on cells at 100 μM or more. At such high concentrations, any hydrophobic molecule could bind multiple targets. As a cell biology reagent, monastrol should be replaced with more potent compounds as soon as they are available. One such reagent is S-trityl cysteine, which is inexpensive, more potent than monastrol and appears to target a similar site in Eg5 [26]. In the meantime, pharmacological SAR of different Eg5 inhibitors is a useful approach to implicate a specific role for Eg5 in biological processes [22].

Monastrol binds a cryptic site that would not have been discovered by virtual screening methods previously employed to find novel small-molecule kinesin inhibitors [31]. It is also notable that this is a flexible drug binding site, which is also the target for the binding of S-trityl cysteine [27] and 3,5-diaryl-4,5-dihydropyrazoles [12], compounds that look very dissimilar in terms of overall shape and charge. While this allosteric site is fortuitous for drug discovery, its evolutionary conservation among vertebrates leads us to speculate that it is required for Eg5 function *in vivo*, either as part of the conformational cycle of the motor domain, or more speculatively by binding an unknown endogenous ligand that could be a protein or a small molecule. Our preliminary small molecule SAR did not yield more potent monastrol derivatives, but highlighted some of the functional groups contributing to Eg5 inhibition. For instance, the specific hydrogen-bonding interaction between Eg5 and the phenol group of monastrol as well as hydrophobic packing of the thiophenol appears crucial for drug binding (Figure 2B), similar to the interactions observed between dihydropyrazoles and Eg5 [12]. A report published during revision of this manuscript described a SAR for monastrol derivatives consistent with our results. However, some inhibitors displaying weak activity in their assays, most notably derivative 2 (Figure 2B) and 4e, were completely inactive in our hands. The discrepancy can be explained by the higher sensitivity of their assay [32].

Although mutations at this site permit microtubule-stimulated ATP hydrolysis by the motor domain of Eg5, their effect on the kinetic parameters K_m_ATP, K0.5MT, k_cat_, and IC_50 _indicate subtle effects on its catalytic cycle. Monastrol decreases the microtubule affinity of Eg5, and one might expect mutations that lower K_0.5_MT to raise IC_50 _for monastrol. This may be true for the L214I, a subtle mutation that would effect transitions between ordered and disordered states of α3 [26]. A surprising result of our mutational analysis showed that mutating R119 greatly elevated K_m_ATP (Figure 4B), indicating a weakened affinity for ATP. In light of a proposed model whereby monastrol stabilizes a pre-ATP hydrolysis conformation of the Eg5 motor domain [23], this merits further investigation using non-equilibrium enzyme kinetics. The mutant L214T, which introduces a hydrophilic residue into a previously hydrophobic environment, lowers k_cat_, as well as overall protein expression (data not shown), as might be expected. Systematic mutational analysis around the monastrol binding sight might identify mutants with discrete effects on microtubule and ATP binding to help dissect the catalytic cycle of kinesin motor proteins.

The monastrol binding site is a promising target for anti-cancer drug development, but our data showing that relatively subtle mutation confer resistance to drug binding without blocking enzymatic function sound a note of caution; Eg5 has potential for sporadic mutation to result in clinical resistance. Pressure to conserve residues at a substrate binding pocket is one explanation for the selectivity of non-competitive inhibitors among related proteins as compared to substrate-competitive inhibitors [33]. The clinical correlate of this biological phenomenon is that non-competitive inhibitors, exemplified by non-nucleoside reverse transcriptase inhibitors (NNRTIs) to treat HIV infection, display fewer non-specific side effects but clinical resistance is easily acquired by mutations in the target protein [34]. Monastrol is a prototype anti-mitotic agent that displays many of the same benefits and limitations of NNRTIs. One obvious concern, if Eg5 inhibitors developed as anti-mitotic drugs also target this site, is the rapid appearance of resistant clones, especially in the context of DNA damaging agents employed clinically. The importance of the monstrol binding site could be explored by gene replacement experiment, swapping drug resistant point mutants for the wild-type protein. Previously such experiments have been hard in vertebrate cells, but recent progress with SiRNA and bacterial artificial chromosome technologies' makes them feasible, even in human cancer cells [35].

## Conclusion

The published crystal structure of monastrol in a ternary complex with the Eg5 motor domain and ADP [25], as well as our own structural studies arrived independently, are consistent with the structure-activity relationship of monastrol against purified, recombinant Eg5 motor domain *in vitro *and in tissue culture cells. Consistent with sequence homology at the drug binding site, we demonstrate the monastrol does not target Klp-61F, the Eg5 homolog in *D. melanogaster*, but arrests *D. rerio *cells in mitosis. Finally, we performed mutational analysis at drug-binding residues which high-lights flexibility at the drug binding site, and suggests a mechanism for genetic resistance to monastrol, and possibly more potent Eg5 inhibitors currently being developed as anti-mitotic agents in cancer chemotherapy.

## Methods

Reagents were obtained from Sigma-Aldrich, unless otherwise indicated. Experimental stocks of taxol (10 mM), PMSF (100 mM), benzimidine hydrochloride (200 mM), 1000 × LPC (10 mg/ml each of leupeptin, pepstatin, and chymostatin), and monastrol derivatives (200 mM) were prepared as DMSO solutions, aliquoted, and stored at -20°C until use. KC25, the standard buffer used in biochemical experiments described below consists of 20 mM PIPES-KOH (pH 6.8), 25 mM KCl. All tissue culture media were supplemented with heat inactivated fetal calf serum (HyClone), penicillin and streptomycin (Cellgro, Mediatech).

### Structural analysis of Eg5-monastrol-ADP complex

We determined the atomic structure of the Eg5-ADP-monastrol complex using multi-wavelength anomalous dispersion to a resolution of 1.80 Å. Although our protein crystals grew under different precipitant conditions than those described by Yan and colleagues, the atomic structure of the Eg5 motor domain were essentially identical to the published results [25]. The atomic coordinates are deposited in the protein database as 1X88. The details of our crystallographic refinement are available as supporting information. Figures in this report were prepared using PyMol [36] based on the structure reported by Yan, et. al. [25], (PDB: 1Q0B).

### Tissue culture assays

Conditions for growing the following cell lines are as described: *D. rerio *AB9 (37), *D. melanogaster *KC167 [38], and BS-C-1 green monkey kidney cells [7]. For immunofluorescence microscopy, cells were grown on 12 mm circular coverslips to 30 % confluence, treated medium supplemented with drug and 0.2% DMSO, or a DMSO alone, for 6 hours, fixed with formaldehyde, and stained for DNA and microtubules, as described [28]. *D. melanogaster *KC167 cells were cultured in Schneider's Drosophila Medium (GIBCO) whereas *D. rerio *AB9 cells and BS-C-1 green monkey kidney cells were cultured at 37°C in Dulbecco's Minimal medium in the presence of 10 % heat inactivated fetal calf serum.

### Cloning, purification, and characterization of Klp-61F motor domain

The motor domain from *D. melanogaster *Klp-61F (amino acid residues 1 to 364) with an engineered C-terminal His_6 _affinity tag was sub-cloned by PCR from a cDNA library (gift of Christine Field) into pRSETa as described [28]. The sequence agreed with the published sequence for the Klp-61F (NP_476818.1 GI:17136642). Expression and purification of the Klp-61F motor domain was performed, as described [28].

### Synthesis of monastrol derivatives

Monastrol derivatives were synthesized via the Biginelli condensation, purified by silica-gel chromatography, and characterized by ^1^H-NMR and tandem liquid chromatography/electrospray ionization mass spectrometry, as described [28]. The ^1^H-NMR resonances in d^6^-DMSO for each derivative, unless previously reported, are listed below:

*Derivative 1*: [ethyl 6-methyl-4-(3-hydroxyphenyl)-2-thioxo-1-methyl-1,2,3,4-tetrahydropyrimidine-5-carboxylate] : 9.79 (s, 1H), 9.44 (s, 1H), 7.11 (t, J = 8.5 Hz, 1H), 6.6 (m, 3H), 5.13 (d, J = 4 Hz, 1H), 4.11 (q, J = 7.5 Hz, 2H), 3.48 (s, 3H), 2.50 (s, 3H), 1.18 (t, J = 7.5 Hz, 3H).

*Derivative 2*: [ethyl 6-methyl-4-(3-hydroxyphenyl)-2-oxo-1,2,3,4-tetrahydropyrimidine-5-carboxylate] : 9.35 (s, 1H), 9.14 (s, 1H), 7.67 (s, 1H), 7.09 (t, J = 8 Hz, 1H), 6.67 (d, J = 7 Hz, 1H), 6.66 (s, 1H), 6.62 (d, J = 7.5 Hz, 1H), 5.06 (d, J = 3 Hz, 1H), 4.00 (q, J = 6.5 Hz, 2H), 2.23 (s, 3H), 1.13 (t, J = 7.5 Hz, 3H).

*Derivative 4a*: [ethyl 6-methyl-4-phenyl-2-thioxo-1,2,3,4-tetrahydropyrimidine-5-carboxylate] : 10.33 (s, 1H), 9.64 (s, 1H), 7.35 (t, J = 7.5 Hz, 2H), 7.28 (t, J = 7 Hz, 1H), 7.22 (d, J = 7 Hz, 2H), 5.17 (d, J = 3.5 Hz, 1H), 4.01 (q, J - 7 Hz, 2H), 2.29 (s, 3H), 1.11 (t, J = 7 Hz, 3H).

*Derivative 4c*: [ethyl 6-methyl-4-(3-fluorophenyl)-2-thioxo-1,2,3,4-tetrahydropyrimidine-5-carboxylate] : 10.41 (s, 1H), 9.69 (s, 1H), 7.41 (dd, J = 8.5, 5.5 Hz, 1H), 7.14 (dd, J= 2, 6.5, 1H), 7.07 (d, J = 8.5 Hz, 1H), 6.98 (d, J = 10 Hz, 1H), 5.20 (d, J = 3.5 Hz, 1H), 4.31 (q, J = 7 Hz, 2H), 2.30 (s, 3H), 1.11 (t, J = 7.5 Hz, 3H).

*Derivative 4d*: [ethyl 6-methyl-4-(3-methoxyphenyl)-2-thioxo-1,2,3,4-tetrahydropyrimidine-5-carboxylate] : 10.33 (s, 1H), 9.63 (s, 1H), 7.27 (t, J = 8 Hz, 1H), 6.85 (d, J = 7 Hz, 1H), 6.78 (d, J = 7.5 Hz, 1H), 6.76 (s, 1H), 5.15 (d, J = 4 Hz, 1H), 4.03 (q, J = 7.5 Hz, 2H), 3.73 (s, 3H), 2.28 (s, 3H), 1.12 (t, J = 7.5 Hz, 3H).

*Derivative 4e*: [ethyl 6-methyl-4-(3-bromophenyl)-2-thioxo-1,2,3,4-tetrahydropyrimidine-5-carboxylate] : 10.41 (s, 1H), 9.68 (s, 1H), 7.50 (d, J = 8.5 Hz, 1H), 7.38 (s, 1H), 7.34 (t, J = 7.5 Hz, 1H), 7.22 (d, J = 7.5 Hz, 1H), 5.17 (d, J = 3.5 Hz, 1H), 4.03 (q, J = 7.5 Hz, 2H), 2.30 (s, 3H), 1.13 (t, J = 7 Hz, 3H).

*Derivative 5a*: [methyl 6-methyl-4-(3-hydroxyphenyl)-2-thioxo-1,2,3,4-tetrahydropyrimidine-5-ketone]: 10.23 (s, 1H), 9.69 (s, 1H), 9.43 (s, 1H), 7.11 (t, J = 7.5 Hz, 1H), 6.6 (m, 3H), 5.19 (d, J = 3.5 Hz, 1H), 2.31 (s, 3H), 2.16 (s, 3H).

*Derivative 5b*: [isobutyl 6-methyl-4-(3-hydroxyphenyl)-2-thioxo-1,2,3,4-tetrahydropyrimidine-5-carboxylate]: 10.30 (s, 1H), 9.60 (s, 1H), 9.42 (s, 1H), 7.12 (t, J = 8Hz, 1H), 6.65 (m, 3H), 5.09 (d, J = 4 Hz, 1H), 3.81 (d, J = 7 Hz, 2H), 2.31 (s, 3H), 1.78 (m, J = 7 Hz, 1H), 0.77 (d, J = 6.5 Hz, 6H).

*Derivative 5c*: [benzyl 6-methyl-4-(3-hydroxyphenyl)-2-thioxo-1,2,3,4-tetrahydropyrimidine-5-carboxylate]: 10.34 (s, 1H), 9.62 (s, 1H), 9.44 (s, 1H), 7.29 (d, J = 5.5, 2H), 7.16 (m, 3H), 7.11 (t, J = 8 Hz, 1H), 6.65 (m, 3H), 5.12 (d, J = 4 Hz, 1H), 5.07 (q, J = 12 Hz, 2H), 2.31 (s, 3H).

*Derivative 6*: [ethyl 6-isopropyl-4-(3-hydroxyphenyl)-2-thioxo-1,2,3,4-tetrahydropyrimidine-5-carboxylate] : 9.74 (s, 1H), 9.59 (s, 1H), 9.45 (s, 1H), 7.13 (t, J = 7.5 Hz, 1H), 6.65 (m, 3H), 5.08 (d, J = 3.5 Hz, 1H), 4.10 (7t, J = 7 Hz, 1H), 4.01 (q, J = 6.5 Hz, 2H), 1.17 (dd, J = 25, 6.5 Hz, 6H), 1.10 (t, J = 6.5 Hz, 3H).

### Preparation of Eg5 motor domain mutants

We used a modified PCR mutagenesis protocol [39] to introduce mutations into hEg5-367H, a plasmid encoding the C-terminally His_6_-tagged motor domain (amino acid residues 1–367) of human Eg5. The wild-type and mutant proteins were expressed and purified using Ni-NTA affinity chromatography and concentrated to between 3 and 10 mg/ml protein as described [28].

### Measurement of ATP hydrolysis kinetics

ATP hydrolysis by recombinantly expressed kinesin motor domains was measured using an NADH-enzyme coupled assay [28]. ATP hydrolysis was measured in KC25 buffer supplemented with 5 μM taxol, 1 mM ATP-KOH (pH 7.0), the appropriate concentration of motor domain, and the appropriate concentrations of taxol-polymerized microtubules. ATP hydrolysis as a function of microtubule concentration was fit to the Michaelis-Menten equation to determine the maximal rate of microtubule-stimulated ATP hydrolysis (k_cat_) and the microtubule concentration for half-maximal stimulation (K_0.5_MT) according to the equation: V = k_cat _× ([MT]/([MT] + K_0.5_MT). A similar analysis, V = V_1 μM _× ([ATP]/([ATP] + K_m_ATP), was used to measure K_m_ATP. Here, V_1 μM _was the maximal rate of ATP hydrolysis by hEg5-367H or its mutants at 1 μM microtubule concentration. To measure the potency (IC_50_) of monastrol derivates, we measured ATP hydrolysis in the presence of 1 μM taxol-stabilized microtubules at various drug concentrations and fit the data to the function: V = 1- V_max _([drug]/([drug] + IC_50_).

## Abbreviations

ATP: Adenosine 5'- triphosphate

ADP: Adenosine 5'-diphosphate

DMSO: Dimethyl sulfoxide

LPC: Mixture of leupeptin, pepstatin, and chymostatin

MT: Microtubule

NADH: Beta-Nicotinamide adenine dinucleotide, reduced

NNRTI: Non-nucleotide reverse transcriptase inhibitor

PDB: Protein database

PIPES: Piperazine-1,4-bis(2-ethanesulfonic acid)

PMSF: Phenyl methyl sulfonyl fluoride

SAR: Structure activity relationship

SiRNA: Small inhibitory Ribonucleic Acid

TCEP: Tris (2-carboxyethyl) phosphine

## Authors' contributions

The corresponding author performed all experiments.

## Supplementary Material

Additional File 1 **Atomic structure determination of the Eg5-monastrol-ADP complex using multi-wavelength anomalous dispersion (PDB 1X88). **Materials and methods for determination of Eg5-monastrol-ADP co-crystal structure using selenomethionine-labeled Eg5 motor domain as independent verification of published structure [25]. Click here for file

Additional File 2 Crystallographic data and refinement statistics for structure of Eg5-monastrol-ADP complex (PDB: 1X88). Click here for file
