# Supplementary material for: Small-molecule and mutational analysis of allosteric Eg5 inhibition by monastrol
Source: BMC Chem Biol. 2006 Feb 27;6:2. doi: 10.1186/1472-6769-6-2 (PMC1448180; doi:10.1186/1472-6769-6-2)
Supplement: Additional File 1 — Atomic structure determination of the Eg5-monastrol-ADP complex using multi-wavelength anomalous dispersion (PDB 1X88). Materials and methods for determination of Eg5-monastrol-ADP co-crystal structure using selenomethionine-labeled Eg5 motor domain as independent verification of published structure [25]. [file 1472-6769-6-2-S1.doc]

### **Additional file 1 – Atomic structure determination of the Eg5-monastrol-ADP complex using multi-wavelength anomalous dispersion (PDB 1X88).**

Materials and methods for determination of Eg5-monastrol-ADP co-crystal structure using selenomethionine-labeled Eg5 motor domain as independent verification of published structure [25].

# Cloning and preparation of hEg5-368

The N-terminal 368 amino acids of *H. sapiens* Eg5 were cloned into pRSETa and expressed in BL21 (DE3) cells (Novagen), as described [27]. All of the following steps were performed at 4 ˚C. Lysis buffer (50 mM PIPES-KOH (pH 6.90), 250 mM KCl, 1 mM MgCl_2_, 100 µM ATP-KOH (pH 7.0), 1 mM PMSF, 1 mM benzimidine hydrochloride, 1 x LPC, and 0.2 % Tween-20) was added to thawed bacterial pellets and then supplemented with 5 mg hen lysozyme (Boehringer-Mannheim) and 300 µl 2-Mercaptoethanol per 100 ml of lysate. The mixture was stirred for 20 minutes, sonicated on ice, and centrifuged at 10,000 x g for 30 minutes. The supernatant was diluted 1:9 with dilution buffer (lysis buffer lacking KCl and detergent) and loaded onto a SP-sepharose (Pharmacia) resin equilibrated with 1:9 (lysis buffer: dilution buffer). The column was washed with 4 volumes of the equilibration buffer and eluted with dilution buffer supplemented with 200 mM NaCl. Protein containing fractions were combined and mixed with (NH_4_)_2_SO_4_ to a final concentration of 1 M. The solution was loaded onto a Hi-Trap Phenyl sepharose column (Amersham) and eluted with a reverse (NH_4_)_2_SO_4_ gradient from 1 M to 0 M. Eg5-containing fractions were mixed and concentrated to approximately 15 mg/ml protein in a 10,000 MWCO Amicon (Millipore) centrifugal concentrator, as judged by Bradford assay (Biorad) using bovine serum albumin type I as a protein standard, and loaded onto a Superdex 75 (Pharmacia) column equilibrated with KC25 supplemented with 1 mM TCEP, 0.02% NaN_3_, 25 µM ATP-KOH (pH 7.0), and 1 x LPC. Eg5-cointaining fractions were combined, concentrated to 10 mg/ml protein as described above, and supplemented with 1 mM ADP-KOH (pH 7.0) and 1 mM racemic monastrol before flash freezing aliquots in liquid nitrogen.

Preparation of selenomethionine-labeled hEg5-368

Starter cultures of freshly transformed B834 (DE3) methionine auxotroph cells (Novagen) were grown in Luria-Bertani broth containing 100 mg ampicillin per liter. At OD600 = 0.9, cells cultures were pelleted, resuspended in labeling medium (1 x M9 minimal medium, 2 mM MgSO_4_, 0.1 mM CaCl_2_, 0.2 % glucose, 0.5 mg/l thiamine hydrochloride, 100 mg/l ampicillin, and 2 mg/l of each amino acid except glycine, alanine, proline, glutamine, asparagine, methionine, and cysteine) and shaken at 37 ˚C. At OD600 = 0.6, each liter of culture was enriched with labeling mix (4 g glucose, 100 mg (L)-selenomethionine, 100 mg each of threonine, lysine hydrochloride, valine, and 50 mg each of leucine, valine, and isoleucine, then and shaken for an additional 10 minutes before induction with 50 mg IPTG. Cultures were then shaken 4 hours at 37 ˚C, pelleted, and flash-frozen in liquid nitrogen. Purification of selenomethionine-labeled hEg5-368 was performed as described above for the unlabeled protein. Mass spectrometric analysis revealed > 95% labeling of the Eg5 motor domain and indicated the N-terminal 9 amino acid residues of the motor domain had been removed by proteolysis.

Protein crystallography

In our preliminary experiments, racemic monastrol inhibited the crystallization of Eg5. We identified novel crystallization conditions for Eg5 in the presence of racemic or enantiomerically pure (S), the ‘active’ stereoisomer, but not (R) -monastrol. Freshly thawed aliquots of frozen hEg5-368 were mixed 1:1 with solution 1 (0.2 M (NH_4_)_2_ tartrate, 20 % PEG-3350). Clustered needles that appeared in 2 µl hanging drops were harvested into solution 1, crushed, and used to microseed 2 µl hanging drops set up with solution 2 (0.2 M (NH_4_)_2_ tartrate, 17 % PEG-3350). Hexagonal plates appearing overnight were grown for one week before harvesting into solution 1 supplemented with 100 µM ADP-KOH (pH 7.0), 100 µM MgCl_2_, and 35 µM (S)-monastrol. Protein crystals were incrementally transferred to a cryosolvent mixture containing 8 % (S,S)-2,3 -butanediol for native protein or 20 % glycerol for labeled protein. X-ray diffraction data for the native (wavelength = 0.99190 Å) and for the selenium labeled (wavelength = 0.97970 Å, 0.97940 Å, and 0.96410 Å) protein was collected at the Advanced Light Source (Lawrence Berkeley National Laboratory) HHMI beamline 8.2.2 and scaled using HKL2000 [41]. The space group is P2_1_2_1_2_1_, a= 69.71 Å, b= 79.65 Å, c= 159.45 Å for the native crystal and a= 69.57 Å, b= 79.76 Å, c= 159.62 Å for the selenomethionine-labeled crystal.

Experimental phases and a preliminary electron density map were obtained from the selenium-MAD data set. The program SOLVE [42] identified 12 of 14 selenium peaks. A model of Eg5 and ADP was built into the density and used as a basis for density modification with automated phase extension of the high-resolution native data set [43]. Atomic coordinate, topology, parameter files for monastrol were generated PRODRG [44]. Refinement of the crystal structure was accomplished using CNS [45], with 10 % of reflections reserved for R-free calculations, via successive rounds of manual rebuilding into composite-omit maps in O [46], simulated annealing, automated water picking, and energy minimization X-PLOR [47]. In the two Eg5 motor domains per asymmetric unit, residues 18 to 362 of domain A and 18 to 366 of domain B are visible, with the exception of 16 amino acids in loop 11 (271 to 287), which are unstructured. Each motor domain is bound to (S)-monastrol, ADP, and a magnesium ion. The refinement statistics are presented, see Additional file 2. Atomic coordinates and structure factors are deposited in the Protein Data Base as 1X88.pdb.

References

41. Z. Otwinowski and W. Minor: **Processing of X-ray** **Diffraction Data Collected in Oscillation Mode.***Meth Enzymology* 1997, Volume 276, part A: 307-326.

42. Terwilliger, T.C. and J. Berendzen: **Automated MAD and MIR structure solution.** *Acta Cryst D* 1999, 55: 849-861.

43. COLLABORATIVE COMPUTATIONAL PROJECT, NUMBER 4: **The CCP4 Suite: Programs for Protein Crystallography.** *Acta Cryst D* 1994, 50: 760-763.

44. A. W. Schuettelkopf and D. M. F. van Aalten: **PRODRG - a tool for high-throughput crystallography of protein-ligand complexes.** *Acta Cryst D* 2004, 60: 1355--1363.

45. Brunger AT, Adams PD, Clore GM, DeLano WL, Gros P, Grosse-Kunstleve RW, Jiang JS, Kuszewski J, Nilges M, Pannu NS, Read RJ, Rice LM, Simonson T, Warren GL: **Crystallography & NMR system: A new software suite for macromolecular structure determination.** *Acta Cryst D* 1998, 54: 905-21.

46. T.A. Jones, M. Bergdoll & M. Kjeldgaard: *O: A macromolecular modeling environment. In: Crystallographic and Modeling Methods in Molecular Design.* Edited byC. Bugg and S. Ealick. Springer-Verlag Press  1990, 189-195.

47. A. T. Brunger: *X-PLOR, version 3.1. A system for X-ray crystallography and NMR.* New Haven, Yale University Press 1992.

**Table 1 – Crystallographic refinement parameters**

This table describes the collection and processing of data.
