# Supplementary figures and images for: Small-molecule and mutational analysis of allosteric Eg5 inhibition by monastrol
Source: BMC Chem Biol. 2006 Feb 27;6:2. doi: 10.1186/1472-6769-6-2 (PMC1448180; doi:10.1186/1472-6769-6-2)

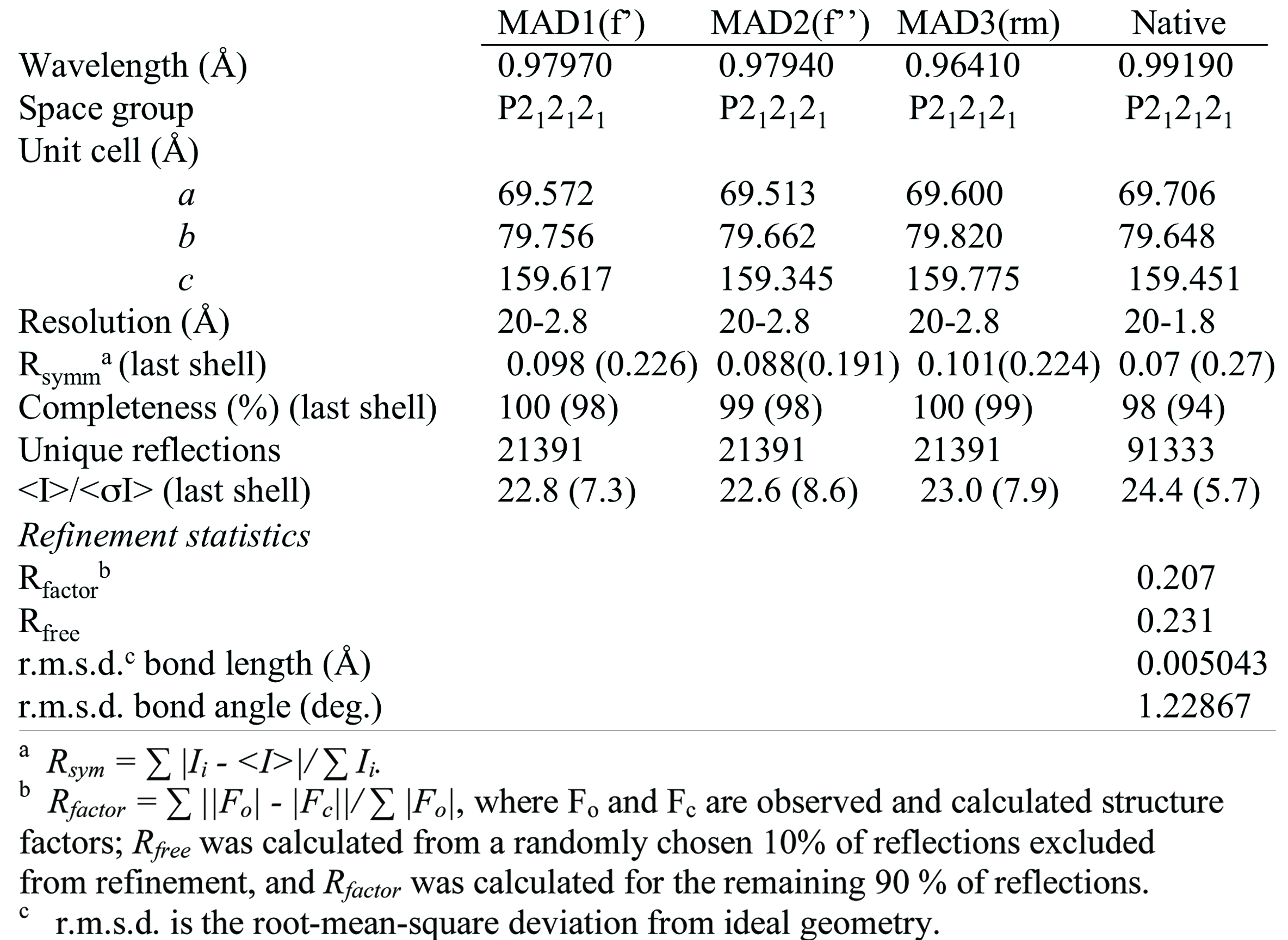

Supplement: Additional File 2 — Crystallographic data and refinement statistics for structure of Eg5-monastrol-ADP complex (PDB: 1X88). [file 1472-6769-6-2-S2.tiff]
